# A Rare Variant of Gastric Adenocarcinoma Presenting as a Symptomatic Early-Stage Submucosal Tumor in the Gastric Antral Primary

**DOI:** 10.7759/cureus.53114

**Published:** 2024-01-28

**Authors:** Hema Narlapati, Brendan R Martino, Pedro Manibusan

**Affiliations:** 1 Internal Medicine, Tripler Army Medical Center, Honolulu, USA; 2 Gastroenterology, Tripler Army Medical Center, Honolulu, USA

**Keywords:** endoscopic ultrasound (eus), gastric malignancy, rare gastric tumor, confocal laser endomicroscopy, locally advanced, submucosal gastric adenocarcinoma

## Abstract

Gastric adenocarcinomas are a well-known malignancy, with the vast majority presenting as primary mucosal invasions. However, a rare form of this cancer presents from the submucosal layer and mimics submucosal tumors (SMTs). This variant of gastric adenocarcinoma is not only rare, but it is also frequently misdiagnosed as other conditions such as gastrointestinal stromal tumors, lymphoma, or sarcoma. This case report describes a unique case of early gastric adenocarcinoma that presented as a submucosal tumor without invasion into the muscularis propria or primary involvement from the gastric mucosa. Additionally, this raises an important clinical question of whether this variant of gastric adenocarcinoma behaves differently from mucosal-origin cancers in terms of invasion and metastasis. This case highlights the diagnostic challenges and the importance of early detection and accurate diagnosis of this rare presentation of gastric adenocarcinoma. This case also provides valuable insights into the clinical variability of submucosal gastric adenocarcinomas and the need for further research to optimize its management and improve patient outcomes.

## Introduction

Gastric adenocarcinomas are a common malignancy affecting millions of people globally, with distinct presentations and histological variants [1]. Submucosal gastric adenocarcinomas, on the other hand, are a rare form of gastric adenocarcinoma accounting for less than 1% of all cases [2]. This variant of gastric cancer presents unique diagnostic and therapeutic challenges due to its anatomical location and variable clinical manifestations. When a submucosal tumor is presented on endoscopy, early detection and accurate diagnosis of submucosal gastric adenocarcinomas have been shown to improve patient outcomes [3]. Submucosal gastric adenocarcinomas often mimic malignant gastrointestinal stromal tumors, pancreatic rest, lipoma, or lymphoma, among other suspects. Rarely would a mucosal gastric adenocarcinoma present submucosally. The challenge presented by avoiding misdiagnosis with submucosal gastric adenocarcinoma can lead to not only a delay in diagnosis from poor early detection but also a potentially poor prognosis if there is metastatic spread [3]. Furthermore, due to the rarity of submucosal gastric adenocarcinomas, there is a paucity of literature on its optimal management and prognosis. Endoscopic ultrasonography (EUS) and biopsy remain the cornerstone of diagnosis for submucosal tumors. Oftentimes, hyperechoic versus hypoechoic features on EUS can help narrow the differential for submucosal tumors [4,5]. Surgical or fine needle aspiration biopsy provides a definitive diagnosis if the sample size is sufficient without high necrotic tissue burden to differentiate submucosal gastric adenocarcinoma from other submucosal tumors. Surgical resection remains the definitive treatment for patients' early-stage submucosal gastric adenocarcinoma [4,5]. Additionally, improving advanced imaging modalities with endoscopic ultrasound may be important to overcome the challenge of misdiagnosing a mucosal adenocarcinoma presenting as a submucosal tumor. 

## Case presentation

A 34-year-old, active-duty Caucasian male with a history of chronic gastroesophageal reflux disease (GERD) presented from Guam with a six-month history of abdominal cramping, anorexia, weight loss, and worsening GERD despite being on the maximum dose of esomeprazole. He had previously failed multiple trials of pantoprazole and omeprazole. Initial computed tomography (CT) in Guam revealed a partially enhancing, approximately 3.4 cm lesion within the gastric antrum. He then underwent an esophagogastroduodenoscopy (EGD) with gastric biopsies. The mucosal biopsy results were negative for malignancy or H. pylori. He was then transferred to Tripler Army Medical Center in Honolulu for further workup of his submucosal mass and chronic symptoms.

**Figure 1:**
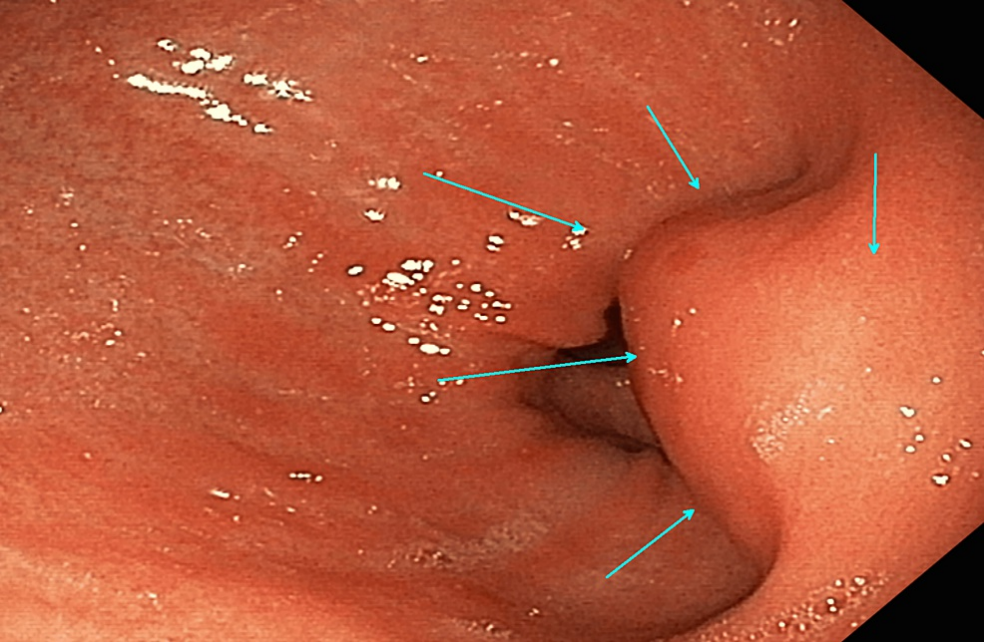
Nonulcerative gastric antral mass (light blue arrows) causing partial obstruction of the gastric outlet

**Figure 2:**
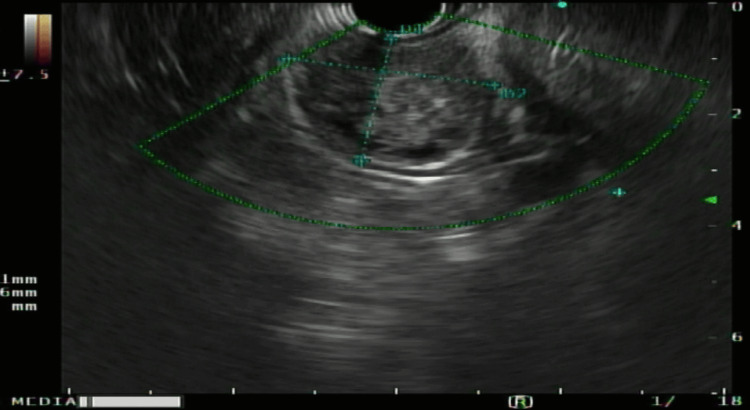
EUS revealing a 3.3 cm submucosal mass with areas of hypodensity suggestive of focal necrosis

The patient underwent repeat EGD and EUS to identify the submucosal mass further. On EGD the gastric antral mass appeared nonulcerative and was causing partial obstruction of the gastric outlet as seen in Figure 1. Furthermore, EUS revealed approximately a 3.3 cm mass that was heterogeneously enhancing and contained multiple hypoechoic densities suggestive of focal necrosis within the submucosal layer (Figure 2). Endoscopic forcep pressure was applied to the mass and showed no surface indentation (negative pillow sign), highly suggestive of malignancy (Figure 1). Fine needle aspiration (FNA) biopsy was performed during the EUS with a 22-gauge endoscopic needle, with specimens obtained for pathologic diagnosis.

Based on endoscopic evaluation of the submucosal mass without mucosal involvement, the leading diagnosis was suspected to be a malignant GI stromal tumor (GIST). However, a histologic review of the FNA samples surprisingly revealed a well-differentiated mucosal gastric adenocarcinoma. Subsequent positron emission tomography/computed tomography (PET/CT) did not show abnormal hypermetabolic activity outside the mass to suggest the presence of metastatic disease. He then underwent repeat EUS for local staging. The mass was found to be abutting the submucosal-muscularis junction but without penetration into the muscularis propria. Investigation of the local lymph nodes showed no pathologic endoscopic character. Given the lack of mucosal involvement, the mass was staged as T1bN0M0 per American Joint Committee on Cancer (AJCC) guidelines, and he was referred to medical and surgical oncology for possible neoadjuvant chemotherapy and surgical resection with distal vs. total gastrectomy.

**Figure 3:**
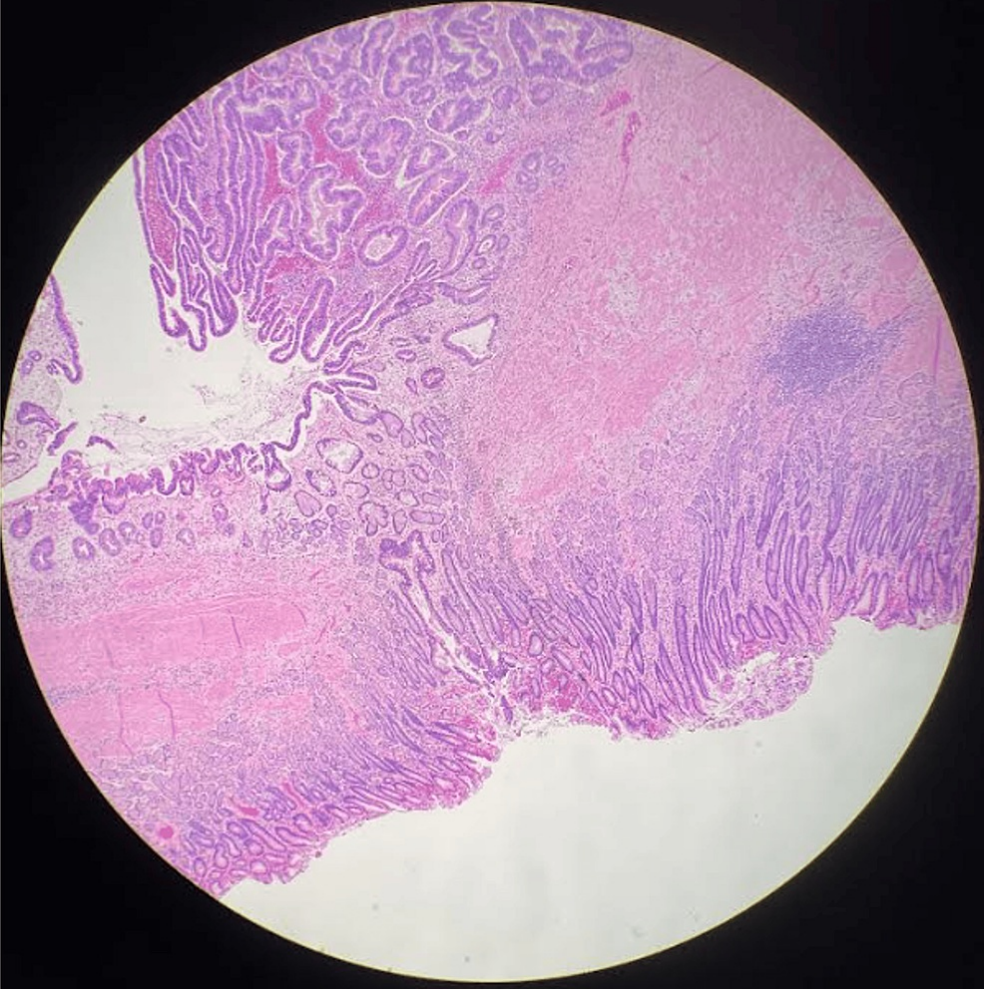
Surgical pathology specimen revealing focal area of surface dysplasia with cells invading the submucosa with intact muscularis propria. Histology confirms a grade 2 moderately differentiated, mixed carcinoma of discrete glandular and signet-ring morphology with poorly cohesive cellular components.

Furthermore, he was then sent for genetic testing to assess for a genetic link to his submucosal gastric adenocarcinoma. A total of nine genes were evaluated including, BRCA1, BRCA2, CDH1, PALB2, PTEN, STK11, TP53, ATM, CHEK2, and p53, all of which were found to be negative. Given that the CDH1 mutation testing was negative for hereditary diffuse gastric cancer (HDGC), the decision was made to proceed with distal subtotal gastrectomy with D2 lymphadenectomy. Surgical pathology confirmed a 3.3 cm tumor with invasion through the submucosal layer (Figure 3). Furthermore, it also revealed an intact muscularis propria layer and a focal area of surface dysplasia. Approximately 27 lymph nodes were dissected, which were negative for metastatic disease. There was no evidence of mesenteric metastases. Histology confirmed a grade 2, moderately differentiated mixed adenocarcinoma with both discrete glandular and signet-ring morphology with poorly cohesive cellular components (Figure 3). Pathological staging was consistent with pT1bN0M0 disease. He continued an uncomplicated postoperative course, with improvement in his obstructive and reflux symptoms.

## Discussion

The majority of gastric adenocarcinomas present as mucosal lesions and are often caught via endoscopic biopsy. However, gastric adenocarcinoma presenting as submucosal neoplasm is much less common. There is much more to be uncovered to understand how the two may differ. Submucosal presentation of gastric adenocarcinoma is a rare occurrence with only a few documented cases in the literature with an incidence rate between 0.2% to 0.6% [2,3,6]. Submucosal gastric adenocarcinoma poses a diagnostic challenge, as it can be often missed in a routine EGD and often requires endoscopic ultrasound for identification and diagnosis. Given its rarity, submucosal gastric adenocarcinomas may be misdiagnosed as other more common submucosal lesions like smooth muscle tumors, stromal tumors, lymphoma, or heterotopic pancreas. This points to the importance of not only widening differentials for early submucosal gastric cancer but also achieving adequate tissue sample size so that pathological diagnosis may render confirmation. Moreover, given the low incidence rate, there is more to uncover from this variant and if the behavior truly varies from the more common mucosal gastric adenocarcinoma. If submucosal gastric adenocarcinoma does behave differently, it is important to determine, in the future, whether treatment options should vary from mucosal gastric adenocarcinoma. The pathophysiology to explain submucosal gastric adenocarcinoma is not fully understood in the literature. However, a logical explanation may be from invasion through the mucosal layer versus vascular seeding. With the first hypothesis, prior micro-damage from repetitive erosion due to inflammatory processes like ethanol and cigarette smoking may be facilitators for gastric adenocarcinoma to present submucosally [7]. As for the second postulation, given that the lymphatic vessels draining the mucosa and muscularis empty into the submucosal network of collecting lymphatics, this may have aided neoplastic cells to appear within the submucosal layer [8]. This further begs the question of whether staging is truly T1a or if further differentiation of staging is required.

On the contrary, it may very well be that this mucosal tissue may have embryonically remained heterotopic within the gastric submucosa. If this is the case, then over time, this tissue may have become metaplastic to adapt to submucosal tissue function. Sequentially speaking, since metaplasia is histologically the precursor to low-grade dysplasia which could result in high-grade dysplasia, the successive functional variation of mucosal tissue may serve as neoplastic precursors [8]. If the latter etiology of heterotopic gastric mucosa remains true, on a cellular level, this would mean that the behavior of submucosal variants versus mucosal variants may very well be distinct. If this theory is clinically supported by tissue research, it would imply that cellular-directed therapies like adjuvant or neoadjuvant chemotherapy may be required, especially if the disease is locally advanced, and curative surgical resection may not suffice. This emphasizes the role of more tissue research to compare the two variants, especially since there are no specific guidelines per the National Comprehensive Cancer Network (NCCN) to objectively stage or treat isolated versus locally advanced submucosal gastric adenocarcinomas [9].

There are also many genetic associations linked with the development of gastric adenocarcinoma. There is an estimated four-fold higher lifetime risk of developing gastric adenocarcinoma in BRCA1 mutation carriers [10]. As previously discussed, CDH1 needs to be ruled out in typical mucosal presentations of gastric adenocarcinoma as this is linked with HDGC. NCCN guidelines recommend total gastrectomy vs. distal gastrectomy due to the high risk of recurrence in HDGC [11]. HER2 overexpression has also been found to be a genetic cause of gastric cancer and carries with it a poor prognosis [12]. Further research is needed to elucidate any genetic associations with submucosal gastric cancers and their implications for diagnosis and treatment.

The use of accurate diagnostic modalities for submucosal gastric adenocarcinomas is essential to improve patient outcomes. Confocal laser endomicroscopy (CLE), an advanced endoscopic imaging modality, can provide a cellular-level view of gastrointestinal epithelia that allows for high diagnostic accuracy for neoplastic disease, especially with reduced tissue sampling required. Confocal laser endomicroscopy can resolve diagnostic dilemmas where conventional imaging may be inconclusive. Given that missing early detection of submucosal gastric cancer may lead to an adverse prognosis with the progression of the disease, CLE may prevent later diagnosis due to its remarkable diagnostic accuracy [13].

## Conclusions

Submucosal presentation of gastric adenocarcinoma is a rare occurrence, comprising <1% of reported cases. Possible mechanisms hypothesized are the presence of heterotopic gastric glandular tissue within the submucosa or gastritis cystica profunda. The behavior of submucosal variants versus mucosal variants of gastric adenocarcinoma is not well understood, and there are currently no guidelines on how to objectively stage lesions of purely submucosal origin. The diagnosis of submucosal gastric adenocarcinoma is more difficult given its depth within the gastric wall compared to mucosal gastric adenocarcinoma, and thus, can result in delayed diagnosis and worse prognosis. Further research is needed to uncover possible genetic associations of submucosal gastric cancers and their implications for diagnosis and treatment. Additionally, if large randomized control trials show that other diagnostic methods like CLE may be highly effective in detecting gastric cancer, this may serve as an ancillary modality to aid in earlier detection to prevent later diagnosis and improve prognosis for patients. 
